# Angiogenesis and Lymphangiogenesis of Gastric Cancer

**DOI:** 10.1155/2010/468725

**Published:** 2010-03-29

**Authors:** Yasuhiko Kitadai

**Affiliations:** Department of Medicine and Molecular Science, Hiroshima University Graduate School of Biomedical Sciences, 1-2-3 Kasumi, Minami-ku, Hiroshima 734-8551, Japan

## Abstract

Tumor angiogenesis is the result of an imbalance between positive and negative angiogenic factors released by tumor and host cells into the microenvironment of the neoplastic tissue. The stroma constitutes a large part of most solid tumors, and cancer-stromal cell interactions contribute functionally to tumor growth and metastasis. Activated fibroblasts and macrophages in tumor stroma play important roles in angiogenesis and tumor progression. In gastric cancer, tumor cells and stromal cells produce various angiogenic factors, including vascular endothelial growth factor, interleukin-8, platelet-derived endothelial cell growth factor, and angiopoietin. In addition, *Helicobacter pylori* infection increases tumor cell expression of metastasis-related genes including those encoding several angiogenic factors. We review the current understanding of molecular mechanisms involved in angiogenesis and lymphangiogenesis of human gastric cancer.

## 1. Introduction

Gastric cancer is the world's second leading cause of cancer death [1]. In Asian countries such as Korea and China, gastric cancer is the leading cause of cancer death. Conventional therapies for advanced-stage gastric cancer include surgery, chemotherapy, and radiotherapy, but the prognosis for advanced-stage disease remains poor. Novel therapeutic strategies are needed, but their development depends on understanding cancer biology, especially changes that occur on the molecular level. A large number of genetic and epigenetic alterations in oncogenes and tumor suppressor genes as well as genetic instability determine the multistep process of gastric carcinogenesis [2]. In addition, the molecular events that characterize gastric cancer differ, depending on the histologic type, whether intestinal- or diffuse-type gastric cancer [2].

**Figure 1:**
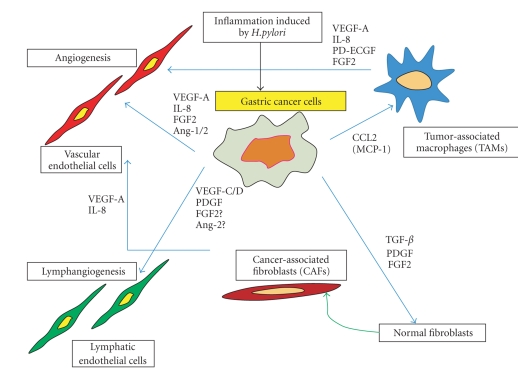
Interaction between gastric cancer cells and stromal cells influences angiogenesis and lymphangiogenesis through various angiogenic factors and cytokines.

Tumor tissue, including gastric cancer, consists of both tumor cells and stromal cells. Tumor growth and metastasis are determined not only by tumor cells themselves but also by stromal cells. Recent studies have shown that interactions between tumor cells and activated stromal cells create a unique microenvironment that is crucial for tumor growth and metastasis (Figure 1) [3, 4]. The organ-specific microenvironment can influence the growth, vascularization, invasion, and metastasis of human neoplasms [5]. 

Angiogenesis and lymphangiogenesis are both essential for tumor growth and metastasis. Increased vascularity enhances the growth of primary neoplasms by supplying nutrients and oxygen, and it provides an avenue for hematogenous metastasis [6, 7]. Weidner et al. [8] first reported a direct correlation between the incidence of metastasis and the number and density of blood vessels in invasive breast cancers. Similar studies have confirmed this correlation in gastrointestinal cancers [9–12]. Induction of angiogenesis is mediated by a variety of molecules released by tumor cells as well as host stromal cells [6, 7]. Clinical prognosis depends on whether lymph node metastasis has occurred. The growth of lymphatic vessels (lymphangiogenesis) in the tumor periphery correlates with lymphatic metastasis in cases of gastric cancer [13, 14]. Lymphangiogenesis is regulated by members of the vascular endothelial growth factor (VEGF) family and their receptors. Herein, we discuss the role of angiogenic and lymphangiogenic factors in the growth and metastasis of human gastric cancer. 

## 2. Tumor Angiogenesis in Gastric Cancer

### 2.1. VEGF-A

Gastric cancer cells produce various angiogenic factors. Of these, VEGF (now termed VEGF-A) is considered one of the strongest promoters of angiogenesis of gastrointestinal tumors [15]. VEGF-A is released by cancer cells. Fibroblasts and inflammatory cells in tumor stroma are also sources of host-derived VEGF-A [16]. VEGF-A, also known as vascular permeability factor, is a secreted protein that may, in addition, play a pivotal role in hyperpermeability of the vessels [17]. Several groups of investigators have reported a correlation between VEGF-A expression and microvessel density (MVD) of human gastric cancer [11, 18, 19]. VEGF-A-positive tumors have been shown to have a poorer prognosis than that of VEGF-A-negative tumors [10, 12, 20].

The prognosis of gastric cancer depends on both histologic type and disease stage [21]. Intestinal-type gastric cancer tends to metastasize to the liver in a hematogenous manner. In contrast, diffuse-type gastric cancer is more invasive; dissemination is predominantly peritoneal. Factors responsible for liver metastasis and peritoneal dissemination have not yet been identified, however, we have found that the angiogenic phenotype differs between intestinal-type and diffuse-type gastric cancers [11, 19]. In comparison to diffuse-type gastric cancer, the intestinal-type is more dependent on angiogenesis. Intestinal-, but not diffuse-type, tumors have been shown to express high levels of VEGF-A, and the level of VEGF-A expression correlates significantly with vessel count [11, 19]. In contrast, fibroblast growth factor (FGF)-2 expression is higher in diffuse-type tumors, especially scirrhous-type tumors [22]. These findings suggest that VEGF-A promotes angiogenesis and progression of human gastric cancers, especially those of the intestinal-type.

Peripheral blood VEGF-A levels, that is, serum and/or plasma concentrations of VEGF-A, have been examined in patients with malignant disease. Ohta et al. [23] examined VEGF-A levels in peripheral blood and the tumor drainage vein and then evaluated these in relation to clinicopathologic features of gastric cancer. They found that the peripheral blood plasma VEGF-A level is increased in patients with venous invasion and that the increase correlates with lymph node metastasis. The level of VEGF-A in plasma from peripheral veins is a sensitive marker for the progression of gastric cancer.

### 2.2. Interleukin (IL)-8

IL-8 is a multifunctional cytokine that can stimulate division of endothelial cells. IL-8 can induce migration of some tumor cells [24] and has been implicated in the induction of angiogenesis in such diverse diseases as psoriasis and rheumatoid arthritis and in some malignant diseases. IL-8 is a known angiogenic factor present in human lung cancer [25, 26] and is also produced by melanomas [27] and bladder [28] and prostate [29] cancers. We examined expression of IL-8 in human gastric cancer and found that most tumor tissues express IL-8 at levels higher than those in the corresponding normal mucosa [29, 30]. The IL-8 mRNA level in neoplasms correlates strongly with vascularization, suggesting that IL-8 produced by tumor cells regulates neovascularization. To provide evidence for a causal role of IL-8 in angiogenesis and tumorigenicity of human gastric cancer, we introduced the IL-8 gene into several human gastric cancer cell lines. Gastric cancer cells transfected with the IL-8 gene and injected orthotopically into the gastric wall of nude mice produce fast-growing, highly vascular neoplasms [31]. 

Gastric cancer cells express not only IL-8 but also IL-8 receptor A (CXCR1) and IL-8 receptor B (CXCR2) [32]. In vitro treatment of human gastric cancer cells (MKN-1 cells) with exogenous IL-8 enhances the expression of epidermal growth factor receptor (EGFR), matrix metalloproteinase (MMP)-9, VEGF-A, and IL-8 mRNAs. In contrast, such treatment decreases expression of E-cadherin mRNA. IL-8 treatment increases the invasive capacity of gastric cancer cells, and IL-8 expression is associated with MMP-9 activity. Collectively, these findings indicate that human gastric cancer cells express receptors for IL-8 and that IL-8 may have autocrine/paracrine roles in the progressive growth of human gastric cancer.

The prognosis for patients with gastric cancer expressing high levels of IL-8 and VEGF-A is significantly poorer than that for patients whose tumors express low levels [20]. A high level of IL-8 in the drainage vein of gastric cancer is associated significantly with a relatively short disease-free survival period [33].

### 2.3. Platelet-Derived Endothelial Cell Growth Factor (PD-ECGF)

PD-ECGF, an endothelial cell mitogen that was initially purified to homogeneity from human platelets, has chemotactic activity for endothelial cells in vitro and is angiogenic in vivo [34]. PD-ECGF was shown to be identical to thymidine phosphorylase, an enzyme involved in pyrimidine nucleoside metabolism [35]. PD-ECGF expression is elevated in several types of solid tumor including colon cancer [36, 37]. We reported that PD-ECGF is associated with angiogenesis of human colon cancer [38]. PD-ECGF is expressed at high levels in vascular tumors that express low levels of VEGF-A [38]. In such colon cancers, the major source of PD-ECGF is the infiltrating cells. A positive association between PD-ECGF expression and MVD has also been reported for human gastric cancer [39–41]. In human gastric cancer, PD-ECGF is expressed more frequently in infiltrating cells than in tumor epithelium [39]. An association exists between PD-ECGF expression by infiltrating cells, VEGF-A expression by tumor epithelium, and vessel counts in intestinal-type gastric cancer but not in diffuse-type gastric cancer [39]. 

### 2.4. Angiopoietin

The angiopoietin family growth factors have been identified as ligands for Tie-2. Angiopoietin-1 activates Tie-2, leading to receptor autophosphorylation upon binding, and it simulates endothelial cell migration in vitro and contributes to blood vessel stabilization by recruitment of pericytes [42]. Angiopoietin-2 is a natural antagonist for Tie-2 receptor [43]. As such, it antagonizes angiopoietin-1-vessel maturation and regulates blood vessel growth, regression, or sprouting, depending on the presence of VEGF [43]. VEGF-A/VEGFR2 is mainly involved in the initiation of angiogenesis, whereas the angiopoietin/Tie2 system is related to remodeling and maturation of vessels [44]. Angiopoietin-1 and -2 are reported to be highly expressed in human gastric cancer [45, 46]. Inhibition of angiopoietin-1 by antisense expression vector was shown to reduce tumorigenesis and angiogenesis of gastric cancer xenografts in nude mice [47]. Production of angiopoietin-2 also contributes to tumor angiogenesis of gastric cancer in the presence of VEGF by induction of proteases in endothelial cells [48].

## 3. Tumor Lymphangiogenesis in Gastric Cancer

The VEGF family includes VEGF-A, -B, -C, -D, -E, and -F and placental growth factor (PlGF) [49]. VEGF-C and VEGF-D are ligands for VEGF receptor (VEGFR)-3 and VEGFR-2. VEGFR-3 is a tyrosine kinase receptor, that is, expressed predominantly in the endothelium of lymphatic vessels [50]. Skobe et al. [51] described VEGF-C as a lymphangiogenic factor that can selectively induce hyperplasia of the lymphatic vasculature. A significant correlation between lymph node metastasis and VEGF-C expression has been reported in gastric cancer [13, 14]. Onogawa et al. [52] examined expression of VEGF-C and VEGF-D by immunohistochemistry in 140 archival surgical specimens of submucosally invasive gastric cancer. VEGF-C immunoreactivity was associated with lymphatic invasion, lymph node metastasis, and increased MVD. There was no association between VEGF-D immunoreactivity and clinicopathologic features. These results suggest that VEGF-C is a dominant regulator of lymphangiogenesis in early-stage human gastric cancer. 

VEGFRs are expressed by a wide variety of cancer cell lines. VEGF-A and VEGFR-1/2 are coexpressed in a number of cancers, including cancers of the breast [53], prostate [54], colon [55], and pancreas [56, 57], suggesting that VEGF-A directly influences tumor cell growth via an autocrine mechanism. VEGFR-3 has also been detected on several types of malignant cells, although the significance of such expression remains unclear. We recently examined the expression and function of VEGFR-3 in gastric cancer cells [58]. In vitro treatment of gastric cancer cell line KKLS, which expresses VEGFR-3, with the ligand of this receptor, VEGF-C, stimulated cell proliferation and increased expression of mRNAs encoding cyclin D1, PlGF, and autocrine motility factor [58]. Thus, VEGF-C may act in both an autocrine fashion and paracrine fashion to promote angiogenesis and further the growth of human gastric cancer.

Other growth factors are reported to be lymphangiogenic, such as FGF-2 [59] and platelet-derived growth factor (PDGF)-BB [60]. The lymphangiogenic effect of FGF-2 appears to be indirect, that is, it occurs via VEGF-C or -D. We recently found that gastric cancer cells produce PDGF-BB and that dilated lymphatics in the tumor periphery express PDGF receptor-*β* (Kodama et al., unpublished data), suggesting that PDGF-BB is a regulator of lymphangiogenesis in gastric cancer. Angiopoietin-2 is crucial for establishing the lymphatic vasculature. VEGF-C/VEGFR-3 signaling is a key primary proliferation pathway for lymphatic vessels, whereas angiopoietin-2 is important in later remodeling stages [61]. The importance of FGF-2, PDGF-BB, and angiopoietin-2 for lymphatic metastasis of human gastric cancer is still unknown.

## 4. Tumor-Stromal Cell Interaction in Tumor Angiogenesis

Tumor stroma consists of activated fibroblasts (myofibroblasts), smooth muscle cells, endothelial cells, and inflammatory cells, including macrophages. Macrophages migrating to tumor stroma are called tumor-associated macrophages (TAMs). The role of TAMs in tumor progression is complicated. Although activated macrophages may have antitumor activity, tumor cells are reported to escape the antitumor activity of TAMs [62]. It has become clear that TAMs are active players in the process of tumor progression and invasion. Indeed, removal of macrophages by genetic mutation has been shown to reduce tumor progression and metastasis [63]. One important characteristic of macrophages is the potential for angiogenic activity. Activated macrophages produce various factors that induce angiogenesis in wound repair [64] and in chronic inflammatory diseases [65, 66]. Upon activation by cancer cells, TAMs can release diverse growth factors, proteolytic enzymes, and cytokines. In clinical studies, high numbers of TAMs have been shown to correlate with high vessel density and tumor progression [67–69]. We previously reported that TAM infiltration into tumor tissue correlates significantly with tumor vascularity in human esophageal and gastric cancers [67, 68]. Ishigami et al. [69] also found direct associations between the degree of TAM infiltration and depth of tumor invasion, nodal status, and clinical stage in cases of gastric cancer. Macrophage recruitment is mediated by a variety of chemoattractants, including monocyte chemoattractant protein-1 (MCP-1/CCL2) and macrophage inflammatory protein 1*α* (MIP-1*α*/CCL3). Of the CC chemokines, MCP-1 is one of the most potent [70]. We found that MCP-1 produced by tumor cells is associated significantly with macrophage infiltration and malignant behavior, such as angiogenesis, tumor invasion, and lymphatic infiltration [67, 68]. Transfection of the MCP-1 gene into gastric cancer cells was shown to cause strong infiltration of macrophages into tumors and enhanced tumorigenicity and metastatic potential in a mouse orthotopic implantation model [71]. Because activated macrophages produce VEGF-A, IL-8, FGF-2, and PD-ECGF, MCP-1 expressed by gastric cancer cells plays a role in angiogenesis via recruitment and activation of macrophages. 

Activated fibroblasts in cancer stroma are prominent modifiers of tumor progression and are therefore called cancer-associated fibroblasts (CAFs) [72, 73]. CAFs have gene expression profiles that are distinct from those of normal fibroblasts [74], and they acquire a modified phenotype, similar to that of fibroblasts associated with wound healing. Tumor tissue contains abundant growth factors, cytokines, and matrix-remodeling proteins; thus, tumors are likened to wounds that never heal [75]. Although the mechanisms that regulate activation of fibroblasts in tumors are not fully understood, PDGF, transforming growth factor-*β*, and FGF-2 are known to be partly involved [72, 73, 76]. We previously reported that CAFs and pericytes express PDGF receptor, and targeting the PDGF receptor on stromal cells inhibits growth and metastasis of human colon cancer [76, 77]. Therefore, CAFs might serve as novel therapeutic targets in cancer patients.

## 5. *Helicobacter pylori (H. pylori)* Stimulates Angiogenesis of Gastric Cancer

A recent advance in understanding the pathogenesis of gastric cancer is recognition of the role of *H. pylori* in gastric carcinogenesis. *H. pylori* infection is thought to contribute significantly to the pathogenesis of atrophic gastritis, intestinal metaplasia, and peptic ulcer. Epidemiologic studies have indicated that infection with *H. pylori* is a risk factor for gastric cancer, and in 1994 the WHO/IARC classified this bacterium as a definite biologic carcinogen [78]. In addition, inoculation of the stomach of Mongolian gerbils with *H. pylori* was shown to be associated with the occurrence of chronic gastritis, intestinal metaplasia, and adenocarcinoma [79, 80]. A recent study showed that *H. pylori* penetrates normal, metaplastic, and neoplastic epithelia to cause a strong immune-inflammatory response and promote gastric carcinogenesis [81]. *H. pylori* is a potent activator of nuclear factor-kB (NF-kB) in gastric epithelial cells. Activation of NF-kB by *H. pylori* infection induces a variety of cytokines, angiogenic factors, MMPs, and adhesion molecules [82, 83]. The relation between *H. pylori* infection and angiogenesis has been studied increasingly over the past few years. We reported previously that *H. pylori*-infected gastric cancer patients show greater tumor vascularity than that of gastric cancer patients after *H. pylori* eradication [84], suggesting that *H. pylori* infection influences angiogenesis of gastric cancer. Some studies suggested that the cagA-positive *H. pylori* strain plays an important role in tissue remodeling, angiogenesis, cancer invasion, and metastasis [85–87]. Crabtree et al. [85] reported that *H. pylori* infection induces IL-8 production by gastric epithelium. We found that coculture of gastric cancer cells with *H. pylori* induces expression of mRNAs encoding IL-8, VEGF-A, angiogenin, urokinase-type plasminogen activator, and MMP-9 by gastric cancer cells [86]. Wu et al. [87] also reported that *H. pylori* influences expression of VEGF-A and MMP-9 and promotes gastric cell invasion via COX-2- and NF-kB-mediated pathways. Expression of COX-2 was found to correlate significantly with VEGF expression and MVD in gastric cancer [88, 89].

## 6. Antiangiogenic Therapy Against Gastric Cancer

A novel category of anticancer drugs, “molecular-targeted drugs”, has become available. Angiogenesis is considered one of the most important molecular targets for anticancer therapy because it is essential for tumor growth and metastasis. VEGF is one of the most potent angiogenic factors and is expressed in almost all human solid tumors investigated, such as colorectal, esophageal, gastric, lung, breast, renal, and ovarian cancers [9, 12, 15, 90]. In these cancers, expression of VEGF correlates with advanced stage disease and poor prognosis. Therefore, inhibiting VEGF is a rational strategy for treating cancer [91]. Bevacizumab is a humanized monoclonal antibody that targets VEGF. Significant prolonged survival has been reported in patients with metastatic colorectal cancer treated with bevacizumab in combination with cytotoxic chemotherapy [92]. Similar improvement were observed in patients with breast and non-small cell lung cancers. A randomized trial evaluating the efficacy of this agent in patients with gastric cancer (the AVAGAST study) is now being conducted internationally; Japan and Korea are included [93]. Several other strategies targeting the VEGF signaling pathway have been developed, including use of soluble receptors binding directly to VEGF ligand, anti-VEGFR antibodies, and VEGR tyrosine kinase inhibitors. These next-generation targeted agents are being evaluated in early clinical studies [91, 93].

## 7. Future Perspectives

Tumor cells are genetically unstable and biologically heterogeneous, and these are the principal causes of the failure of systemic chemotherapies. It has been believed that endothelial cells and CAFs in tumor stroma are genetically stable and that these cells will not become drug resistant in response to antivascular therapy. However, recent studies showed that endothelial cells in certain tumor vessels are aneuploid and that they express neoplastic markers [94]. Recently, investigators have come to appreciate the significance of other cell types, such as pericytes and vascular smooth muscle cells, that make up vascular structures and are essential for the function and survival of endothelial cells. The structure and function of vessels differ in different tissues and in tumors at different sites. Inhibition of activated stromal cell components including TAMs and CAFs may effectively alter the tumor microenvironment involved in tumor angiogenesis and progression. Understanding the cellular and molecular mechanisms that regulate vascularization of tumors may facilitate development of effective antivascular therapies.
